# The thymoproteasome hardwires the TCR repertoire of CD8^+^ T cells in the cortex independent of negative selection

**DOI:** 10.1084/jem.20201904

**Published:** 2021-02-08

**Authors:** Izumi Ohigashi, Melina Frantzeskakis, Alison Jacques, Sayumi Fujimori, Aya Ushio, Fusano Yamashita, Naozumi Ishimaru, Da Yin, Margaret Cam, Michael C. Kelly, Parirokh Awasthi, Kensuke Takada, Yousuke Takahama

**Affiliations:** 1 Division of Experimental Immunology, Institute of Advanced Medical Sciences, University of Tokushima, Tokushima, Japan; 2 Experimental Immunology Branch, National Cancer Institute, National Institutes of Health, Bethesda, MD; 3 Department of Oral Molecular Pathology, Tokushima University Graduate School of Biomedical Sciences, Tokushima, Japan; 4 Collaborative Bioinformatics Resource, National Cancer Institute, National Institutes of Health, Bethesda, MD; 5 Single Cell Analysis Facility, National Cancer Institute, National Institutes of Health, Bethesda, MD; 6 Transgenic Mouse Model Laboratory, Frederick National Laboratory for Cancer Research, National Cancer Institute, National Institutes of Health, Bethesda, MD; 7 Laboratory of Molecular Medicine, Faculty of Veterinary Medicine, Hokkaido University, Sapporo, Japan

## Abstract

The thymoproteasome expressed specifically in thymic cortical epithelium optimizes the generation of CD8^+^ T cells; however, how the thymoproteasome contributes to CD8^+^ T cell development is unclear. Here, we show that the thymoproteasome shapes the TCR repertoire directly in cortical thymocytes before migration to the thymic medulla. We further show that the thymoproteasome optimizes CD8^+^ T cell production independent of the thymic medulla; independent of additional antigen-presenting cells, including medullary thymic epithelial cells and dendritic cells; and independent of apoptosis-mediated negative selection. These results indicate that the thymoproteasome hardwires the TCR repertoire of CD8^+^ T cells with cortical positive selection independent of negative selection in the thymus.

## Teaser

The thymoproteasome expressed in thymic cortical epithelium optimizes the generation of CD8^+^ T cells. This study reveals that the thymoproteasome hardwires the TCR repertoire of CD8^+^ T cells with cortical positive selection independent of thymic negative selection processes.

## Graphical Abstract

**Figure:**
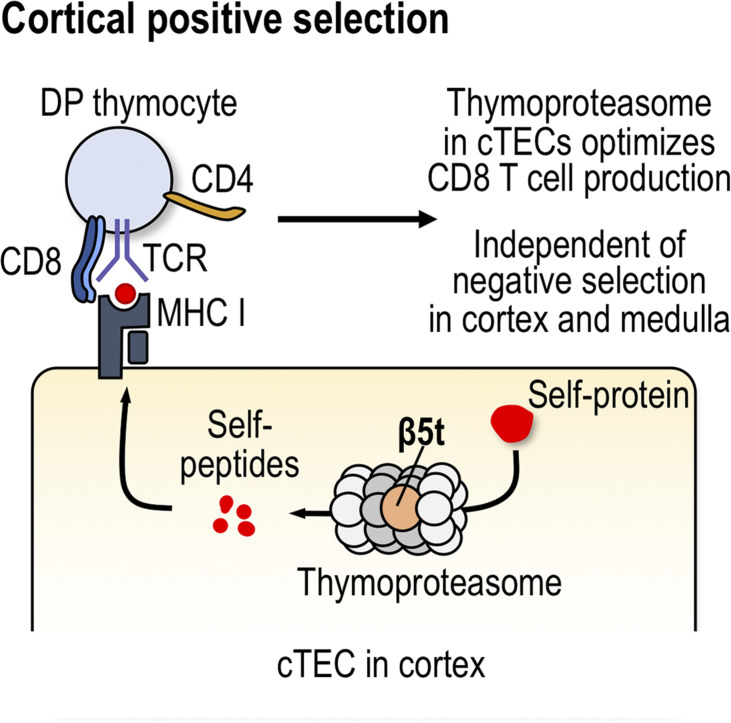

## Introduction

CD8^+^ T cells play an essential role in the protective immune response to malignancy and viral infection. The development of CD8^+^ T cells requires TCR engagement in CD4^+^CD8^+^ immature thymocytes newly generated in the thymic cortex (Starr et al., 2003; Klein et al., 2014). This TCR engagement, referred to as positive selection, is mediated primarily by peptide–MHC class I (MHC-I) complexes displayed by cortical thymic epithelial cells (cTECs; Anderson et al., 1994; Laufer et al., 1996). The majority of cTECs carry unique machinery to produce MHC-I–associated peptides by the specific expression of the thymoproteasome, which contains cTEC-specific proteolytic subunit β5t encoded by *Psmb11* (Murata et al., 2007; Sasaki et al., 2015; Uddin et al., 2017). The β5t-containing thymoproteasome in cTECs is essential for the optimal generation of functionally competent self-protective CD8^+^ T cells (Nitta et al., 2010; Xing et al., 2013; Takada et al., 2015); however, the mechanism for thymoproteasome-dependent CD8^+^ T cell production in the thymus remains unclear. Whether the thymoproteasome directly optimizes cTEC-mediated positive selection or indirectly contributes to T cell production by reducing negative selection has remained controversial.

The thymoproteasome exhibits unique specificity in protein degradation and produces a unique set of MHC-I–associated peptides (Sasaki et al., 2015). It is possible that the thymoproteasome-dependent peptide–MHC-I complexes exclusively expressed by cTECs contribute to the optimization of CD8^+^ T cell production by enhancing positive selection and/or by reducing negative selection (Murata et al., 2018). The thymoproteasome may enhance positive selection by producing MHC-I–associated peptides that are uniquely advantageous to induce positive selection (Xing et al., 2013; Sasaki et al., 2015). An alternative, but not mutually exclusive, possibility is that the thymoproteasome may optimize CD8^+^ T cell production by avoiding and reducing negative selection (Kincaid et al., 2016; Tomaru et al., 2019). The thymoproteasome-dependent self-peptide–MHC-I complexes may create a difference in MHC-I–associated self-peptides between cTECs and other antigen-presenting cells. This difference in self-peptides between cTECs and other thymic antigen-presenting cells provides a window for positively selected CD8^+^ T cells to escape from subsequent negative selection by identical self-peptide–MHC-I complexes displayed by a variety of thymic antigen-presenting cells not limited to cTECs (Kincaid et al., 2016; Tomaru et al., 2019). Indeed, thymocytes that are positively selected in the thymic cortex migrate to the thymic medulla, in which various antigen-presenting cells, including medullary thymic epithelial cells (mTECs) and dendritic cells (DCs), establish self-tolerance in newly generated T cells (Klein et al., 2014; Cosway et al., 2017). It is possible that the thymic medulla contributes to the negative selection of thymocytes that have been positively selected by overlapping self-peptide–MHC-I ligands shared by cTECs and medullary antigen-presenting cells, while allowing the survival and development of CD8^+^ T cells positively selected by a unique set of thymoproteasome-dependent self-peptide–MHC-I ligands expressed in cTECs.

To better understand the mechanism for the thymoproteasome-mediated optimization of CD8^+^ T cell production, we performed a high-throughput deep-sequencing analysis of the TCRαβ repertoire in polyclonal CD8^+^ T cells produced in the presence or absence of the thymoproteasome. We found that the thymoproteasome influences the V(D)J sequences of the TCRα and TCRβ chains in CD8^+^ T cells, and that the thymoproteasome hardwires the TCR repertoire of positively selected thymocytes in the thymic cortex before the migration to the thymic medulla. We further found that the thymoproteasome optimizes CD8^+^ T cell production independent of the thymic medullary microenvironment; independent of additional antigen-presenting cells, including mTECs and DCs; and independent of the apoptosis-mediated negative selection of developing thymocytes. These results demonstrate that the negative selection–dependent mechanism has no role in the thymoproteasome-mediated optimization of CD8^+^ T cells. Instead, our results reveal a direct contribution of the thymoproteasome intrinsic to positive selection in the thymic cortex governing the generation of the TCR repertoire of CD8^+^ T cells.

## Results

### The thymoproteasome affects TCRαβ V(D)J sequences in CD8^+^ T cells

**Figure 1.**
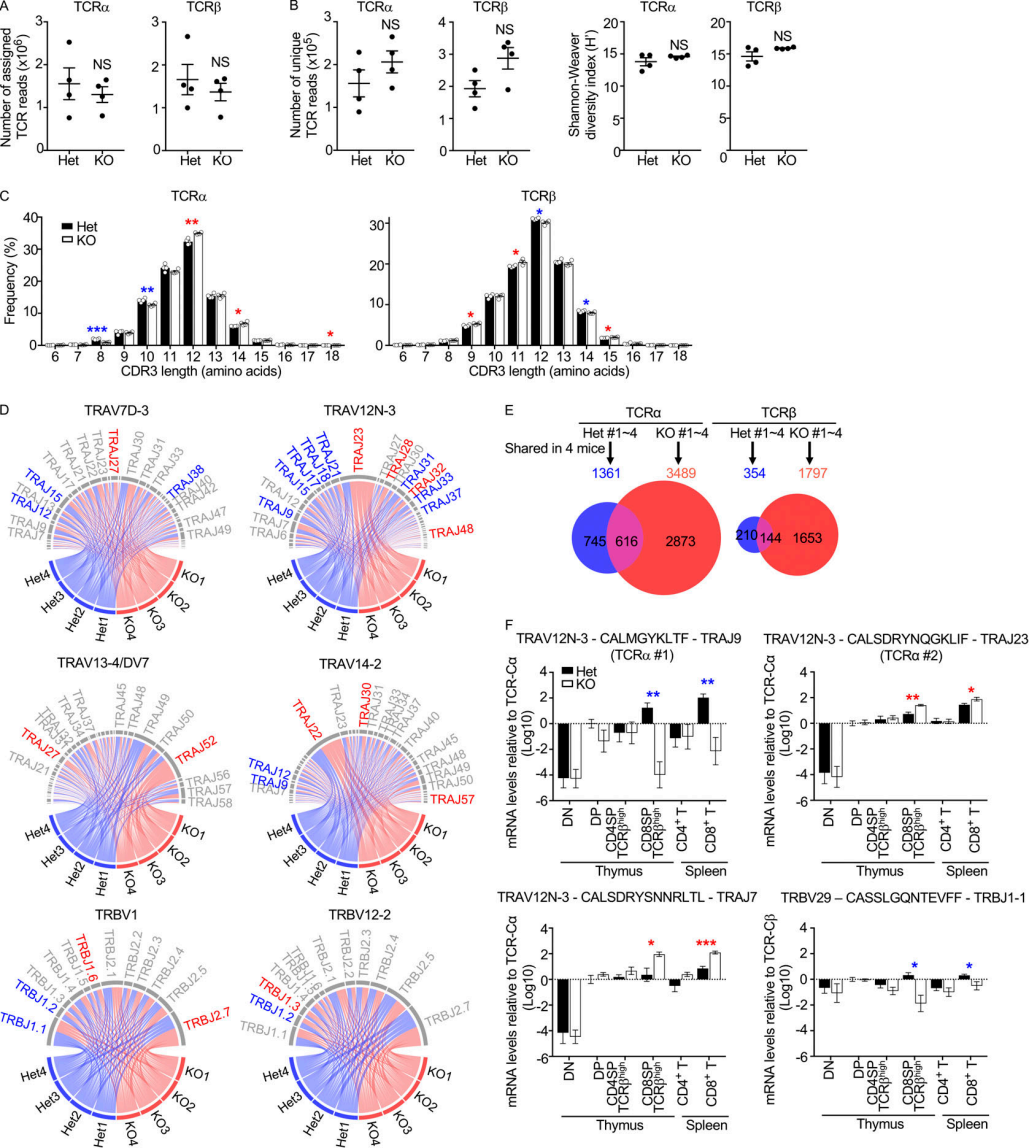
**The thymoproteasome affects V(D)J sequences of TCRα and TCRβ chains in polyclonal CD8^+^ T cells.** **(A)** Numbers (means and SEMs, *n* = 4 in two independent measurements) of TCR sequences assigned to in-frame V(D)J-rearranged TCRs in 10^6^ CD8^+^ T cells from four individual β5t^+/−^ (Het) mice and four individual β5t^−/−^ (KO) mice. **(B)** Numbers (means and SEMs, *n* = 4) of unique TCR reads (left) and Shannon-Weaver diversity indexes of TCR repertoire diversity (right) in CD8^+^ T cells from Het mice and KO mice. **(C)** Frequencies (means and SEMs) of CDR3 length in CD8^+^ T cells from Het mice and KO mice. Statistically significant increases and decreases of frequencies in KO cells are highlighted by red and blue asterisks, respectively. *, P < 0.05; **, P < 0.01; ***, P < 0.001 (by unpaired *t* test). **(D)** Circos plots showing the use of V-J combinations in CD8^+^ T cells from Het mice and KO mice. Statistically significant difference in frequencies between Het (blue) and KO (red) groups is highlighted by letters in blue or red. **(E)** Venn diagrams showing unique and overlapped numbers of TCR full-length sequences shared in all four Het mice (1,361 TCRα genes and 354 TCRβ genes) and all four KO mice (3,489 TCRα genes and 1,797 TCRβ genes). **(F)** Quantitative RT-PCR analysis of mRNA expression levels (means and SEMs, *n* = 7 in seven independent measurements) of indicated TCR full-length sequences relative to TCR-Cα or TCR-Cβ levels in CD4^+^CD8^+^ thymocytes isolated from Het mice. Statistically significant increases and decreases of mRNA levels in KO cells are highlighted by red and blue asterisks, respectively. *, P < 0.05; **, P < 0.01; ***, P < 0.001 (by unpaired *t* test with Welch’s correction for unequal variances).

**Figure S1.**
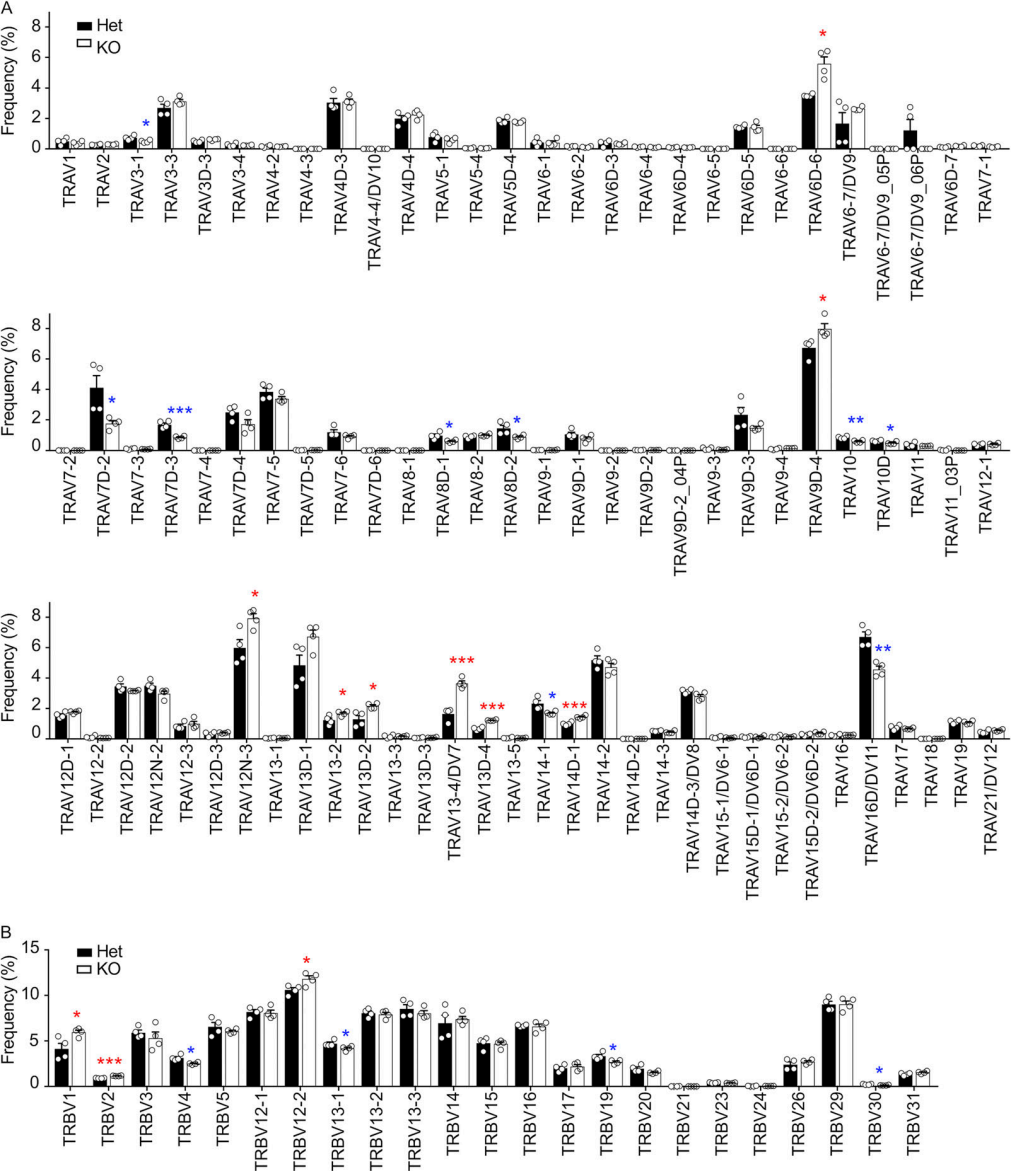
**The use of TCRα and TCRβ V regions detected in CD8^+^ T cells from β5t-deficient mice. (A and B) **Frequencies (means and SEMs) of the use of TCRα V regions (A) and TCRβ V regions (B) detected in CD8^+^ T cells from β5t^+/−^ (Het) mice and β5t^−/−^ (KO) mice. Statistically significant increases and decreases of frequencies in KO cells are highlighted by red and blue asterisks, respectively. *, P < 0.05; **, P < 0.01; ***, P < 0.001 (by unpaired *t* test).

To explore the mechanism of the thymoproteasome-dependent optimization of CD8^+^ T cells, a high-throughput deep-sequencing analysis of TCRα and TCRβ mRNAs was performed to demonstrate the repertoire of TCR–V(D)J sequences expressed in CD8^+^ T cells isolated from four individual β5t-deficient mice and four individual control mice. The number of sequences assigned to in-frame V(D)J-rearranged TCRs exceeded the number of T cells used for the analysis (Fig. 1 A), verifying that the depth of the TCR sequencing analysis was relevant to the size of the actual TCR repertoire (Casrouge et al., 2000) and comparable to the depths in recent TCR repertoire analyses (Lu et al., 2019; Textor et al., 2018). We first noticed that the repertoire of TCRα and TCRβ sequences in CD8^+^ T cells from β5t-deficient mice was similarly diverse compared with that from control mice, as estimated by the Shannon-Weaver diversity index (Fig. 1 B), suggesting that the absence of the thymoproteasome does not diminish, but maintains, the diversity in the TCR repertoire of CD8^+^ T cells. We also found that the MHC-I–associated peptide-interacting CDR3 sequences were longer in TCRα and shorter in TCRβ in CD8^+^ T cells from β5t-deficient mice compared with control mice (Fig. 1 C), indicating that the length distribution of TCRαβ CDR3 sequences in CD8^+^ T cells is different between β5t-deficient mice and control mice. The use of many V regions in TCRα and TCRβ was significantly different (Fig. S1), and the use of V-J combinations was biased between β5t-deficient mice and control mice (Fig. 1 D).

Interestingly, we found that more than 50% of TCRα and TCRβ full-length sequences, including the CDR3 sequences, shared in CD8^+^ T cells isolated from all four mice were distinct between β5t-deficient mice and control mice (Fig. 1 E). Strikingly, many TCRα and TCRβ full-length sequences were detected in CD8^+^ T cells from all four β5t-deficient mice, but not from any control mice, whereas other TCRα and TCRβ full-length sequences were detected in CD8^+^ T cells from all four control mice, but not from β5t-deficient mice (Table S1). Quantitative RT-PCR analysis confirmed that the preferential usage of these TCR V(D)J sequences in either β5t-deficient mice or control mice was detected similarly in peripheral CD8^+^ T cells and in CD4^−^CD8^+^ TCRβ^high^ thymocytes (Fig. 1 F). These results indicate that the thymoproteasome affects the TCR repertoire of the polyclonal CD8^+^ T cell population, influencing the selection of V(D)J full-length sequences of TCRα and TCRβ chains in the thymus.

### The thymoproteasome shapes the TCR repertoire of positively selected thymocytes in thymic cortex before migration to thymic medulla

**Figure 2.**
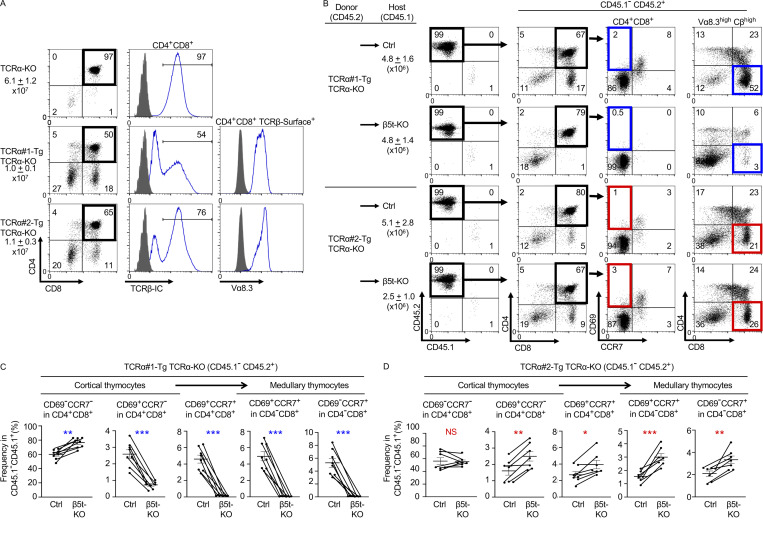
**The thymoproteasome shapes the TCR repertoire of positively selected thymocytes in the thymic cortex before migration to the thymic medulla.** **(A)** Flow cytometric analysis of thymocytes from TCRα-KO mice, TCRα#1-Tg TCRα-KO mice, and TCRα#2-Tg TCRα-KO mice at 6 wk old. Total viable thymocyte numbers (means and SEMs, *n* = 5–7 in three independent measurements) are listed. Shown are CD8 and CD4 profiles of total viable thymocytes (left), intracellular (IC) TCRβ histograms in CD4^+^CD8^+^ thymocytes (middle), and surface Vα8.3 expression in CD4^+^CD8^+^ surface TCRβ^high^ thymocytes (right). Background fluorescence histograms are also shown (shaded). Numbers in plots indicate the frequency of cells within indicated area. **(B–D)** Flow cytometric analysis of thymocytes from bone marrow (BM) chimera mice. BM cells from TCRα#1-Tg TCRα-KO or TCRα#2-Tg TCRα-KO mice (CD45.1^−^CD45.2^+^) were transferred into lethally irradiated β5t-sufficient (control [Ctrl]) or β5t-deficient (KO) mice (CD45.1^+^CD45.2^−^). **(B)** Representative profiles of reconstituted CD45.1^−^CD45.2^+^ thymocytes were gated as indicated and analyzed for cell-surface expression of indicated molecules. Numbers in plots indicate the frequency of cells within the indicated area. **(C and D)** Frequencies (means and SEMs, *n* = 8 in seven independent measurements) of indicated thymocyte subpopulations in CD45.1^−^CD45.2^+^ thymocytes derived from TCRα#1-Tg TCRα-KO (C) and TCRα#2-Tg TCRα-KO (D) BM cells are shown. *, P < 0.05; **, P < 0.01; ***, P < 0.001 (by paired *t* test).

Thymoproteasome-dependent preferential usage of individual TCR sequences during thymocyte development was further examined by generation of mice expressing single and fixed TCRα chains. We focused on TRAV12N-3 - CALMGYKLTF - TRAJ9 (TCRα#1 in Fig. 1 F), which was more frequently used in CD8^+^ T cells from control mice than β5t-deficient mice, and TRAV12N-3 - CALSDRYNQGKLIF - TRAJ23 (TCRα#2 in Fig. 1 F), which was identified to be more frequently used in CD8^+^ T cells from β5t-deficient mice than control mice. We engineered transgenic mice expressing these TCRα chains and then backcrossed onto TCRα-deficient mice to generate mice specifically expressing TCRα#1 or TCRα#2 full-length TCRα chains. Analysis of thymocyte development demonstrated that the majority of transgenic TCRα chains expressed by CD4^+^CD8^+^ thymocytes were associated with TCRβ chains (Fig. 2 A), although the expression of transgenic TCRα chains decreased the generation of CD4^+^CD8^+^ TCRαβ^+^ thymocytes (Fig. 2 A) by impairing the β-selection process, as previously reported (Erman et al., 2002; Baldwin et al., 2005). Interestingly, thymocytes that expressed either TCRα#1 or TCRα#2 preferentially differentiated into CD4^−^CD8^+^ TCRαβ^high^ T cells rather than CD4^+^CD8^−^ TCRαβ^high^ T cells (Fig. 2, A and B), indicating that CDR3-containing TCRα full-length V-J sequence influences CD4/CD8 lineage choice in immature thymocytes.

**Figure S2.**
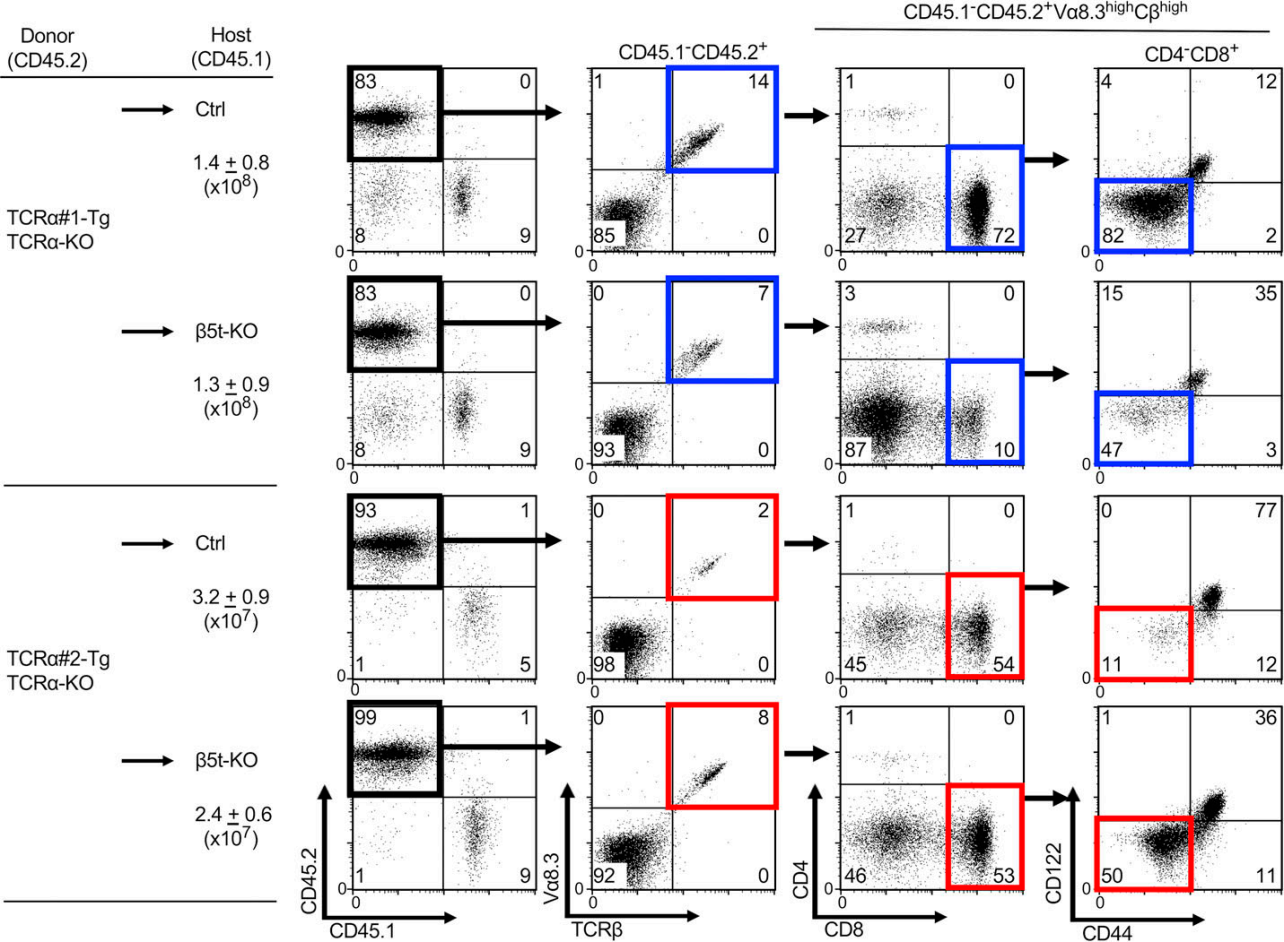
**Flow cytometric analysis of spleen cells from indicated bone marrow chimera mice.** Total viable cell numbers (means and SEMs, *n* = 5–6 in four independent measurements) are listed. Representative profiles of reconstituted CD45.1^−^CD45.2^+^ spleen cells were gated as indicated and analyzed for cell-surface expression of indicated molecules. Numbers in plots indicate frequency of cells within indicated area. Ctrl, control.

More interestingly, we found that thymocytes expressing TCRα#1 generated CD4^−^CD8^+^ TCRαβ^high^ thymocytes and spleen-naive CD8^+^ T cells more efficiently in control thymus than in β5t-deficient thymus (Fig. 2, B and C; and Fig. S2). On the contrary, thymocytes expressing TCRα#2 generated CD4^−^CD8^+^ TCRβ^high^ thymocytes and spleen-naive T cells independent of β5t and preferentially in β5t-deficient thymus than in control thymus (Fig. 2, B and D; and Fig. S2). The difference in the frequency of CD4^−^CD8^+^ thymocytes between β5t-deficient thymus and control thymus was sharper in TCRα#1-expressing thymocytes than TCRα#2-expressing thymocytes (Fig. 2, C and D), in agreement with the sharper difference in TCRα#1 mRNA levels than TCRα#2 mRNA levels in CD8^+^ T cells between β5t-deficient mice and control mice (Fig. 1 F).

**Figure S3.**
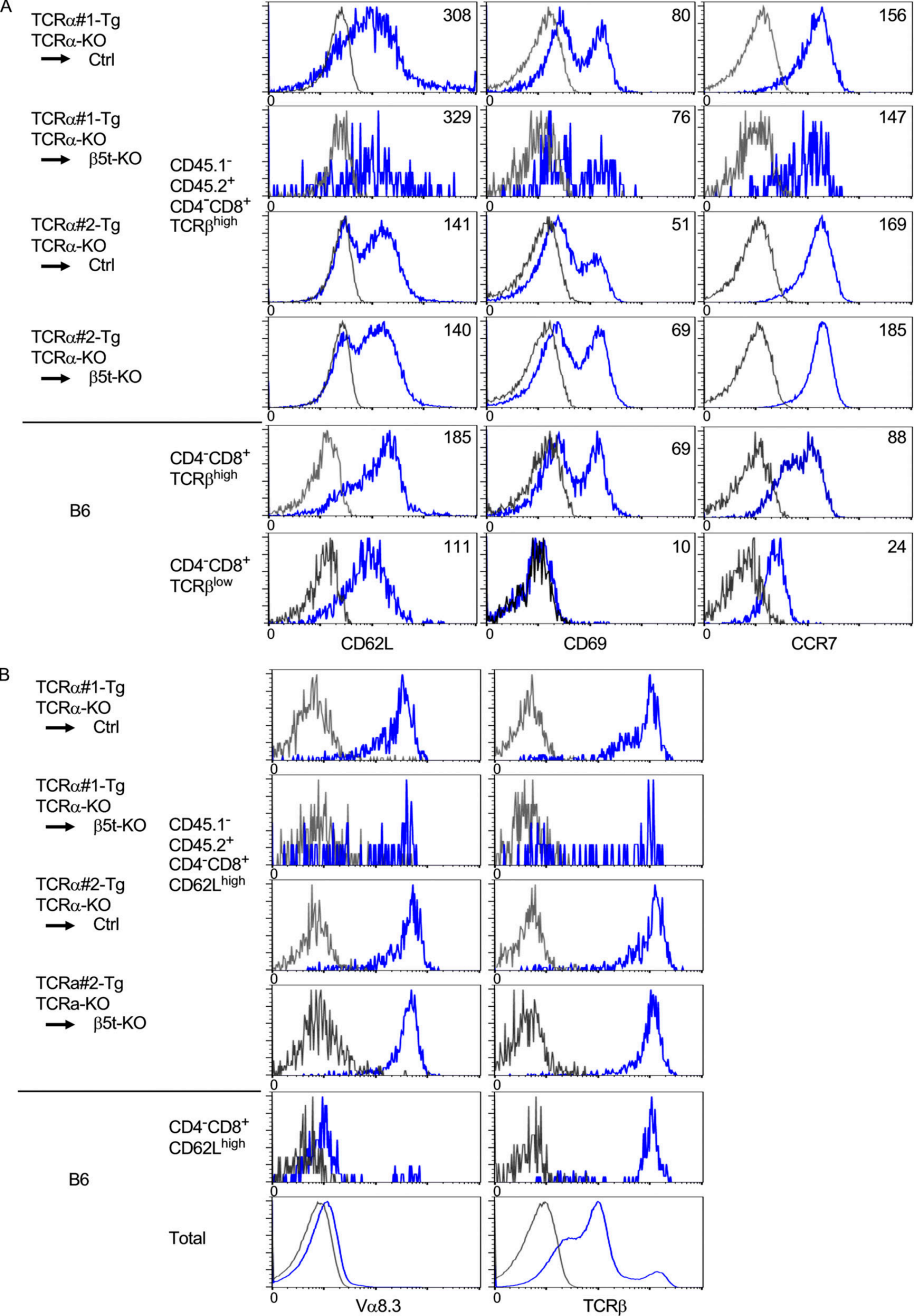
**Flow cytometric profiles of CD62L, CD69, CCR7, Vα8.3, and TCRβ expressed by thymocyte subpopulations from bone marrow chimera mice.(A)** Flow cytometric analysis of CD62L, CD69, and CCR7 expressed by CD4^−^CD8^+^ TCRβ^high^ thymocytes from indicated bone marrow chimera mice and by CD4^−^CD8^+^ TCRβ^high^ and CD4^−^CD8^+^ TCRβ^negative/low^ thymocytes from B6 mice. Numbers in histograms indicate mean fluorescence intensity. **(B)** Flow cytometric analysis of Vα8.3 and TCRβ expressed by CD4^−^CD8^+^ CD62L^high^ thymocytes from indicated bone marrow chimera mice and by CD4^−^CD8^+^ CD62L^high^ and total thymocytes from B6 mice. Background fluorescence histograms are shown in gray. Representative results of *n* = 4 in two independent measurements are shown. Ctrl, control.

The expression profiles of T cell maturation–associated cell-surface molecules, including CD62L, CD69, and CCR7, demonstrated that CD4^−^CD8^+^ TCRβ^high^ thymocytes from the bone marrow chimera mice resembled CD4^−^CD8^+^ TCRβ^high^ mature thymocytes, rather than CD4^−^CD8^+^ TCRβ^low^ immature thymocytes, in normal B6 mice (Fig. S3 A). Furthermore, the surface Vα8.3 expression profiles on CD62L^high^ CD4^−^CD8^+^ thymocytes from TCRα#1- and TCRα#2-transgenic bone marrow chimera mice supported that the vast majority of Vα8.3^high^ CD4^−^CD8^+^ thymocytes detected in these bone marrow chimera mice represented CD4^−^CD8^+^ mature thymocytes rather than CD4^−^CD8^+^ immature thymocytes (Fig. S3 B).

**Figure 3.**
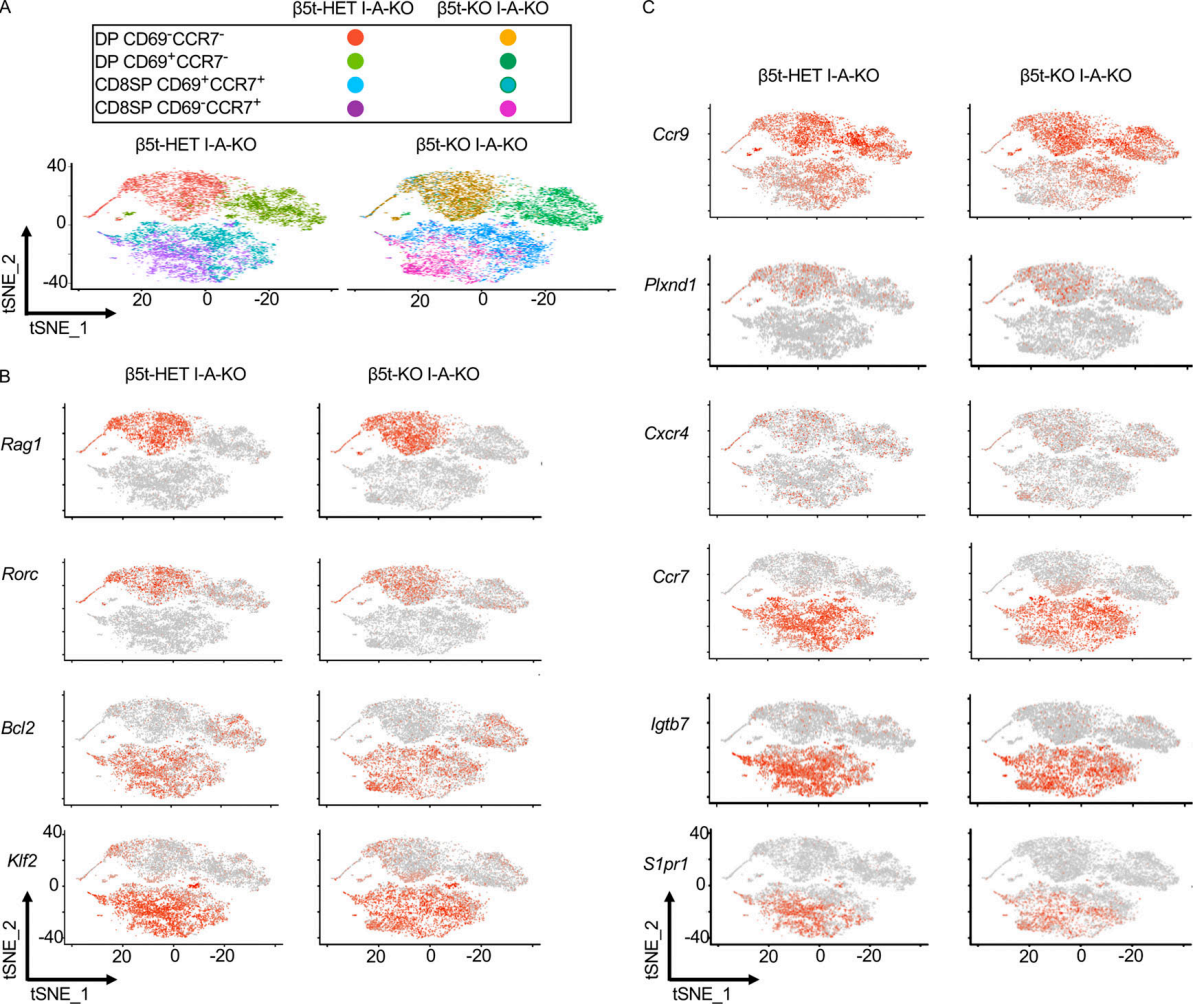
**Single-cell RNA sequencing analysis of thymocyte subpopulations.** **(A)** t-Distributed stochastic neighbor embedding plots of indicated thymocyte subpopulations isolated from β5t-Het I-Aβ-KO and β5t-KO I-Aβ-KO mice at 6 wk old (*n* = 2 in two independent measurements). I-Aβ-KO mice, lacking cell-surface MHC-II expression, were used to specifically detect MHC-I–dependent selection and development of CD4^+^CD8^+^ (DP) immature thymocytes into CD4^−^CD8^+^ mature thymocytes. **(B and C)** Feature plots for indicated genes encoding intracellular molecules (B) and cell-surface molecules (C).

Most importantly, the preferential expression of those TCR sequences in CD4^−^CD8^+^ thymocytes and CD8^+^ T cells in either β5t-deficient mice or control mice was detected as early as the developmental stage at CD4^+^CD8^+^ CD69^+^CCR7^−^ thymocytes (Fig. 2, B–D). This stage of thymocytes represents CD4^+^CD8^+^ thymocytes that have recently received TCR signaling, being CD69^+^, in the thymic cortex before migration to the medulla, which is dependent on the medullary chemokine-derived signaling through CCR7 (Van Laethem et al., 2013; Takahama, 2006). Single-cell RNA sequencing analysis verified that CD4^+^CD8^+^ CD69^+^CCR7^−^ thymocytes in either β5t-deficient mice or control mice (Fig. 3 A) exhibited molecular signatures of TCR-signaled thymocytes (*Rag1*^low^ *Rorc*^low^ *Bcl2*^high^ CD4^+^CD8^+^CD69^+^; Fig. 3 B) that are still localized in the thymic cortex (*Ccr9*^high^ *PlexinD1*^high^ *Cxcr4*^high^ *Ccr7*^low^CCR7^−^ *IntegrinB7*^low^ *Klf2*^low^ *S1pr1*^low^; Fig. 3, B and C). These results indicate that the thymoproteasome directly determines the TCR repertoire of positively selected thymocytes in the thymic cortex before the migration to the thymic medulla.

**Figure S4.**
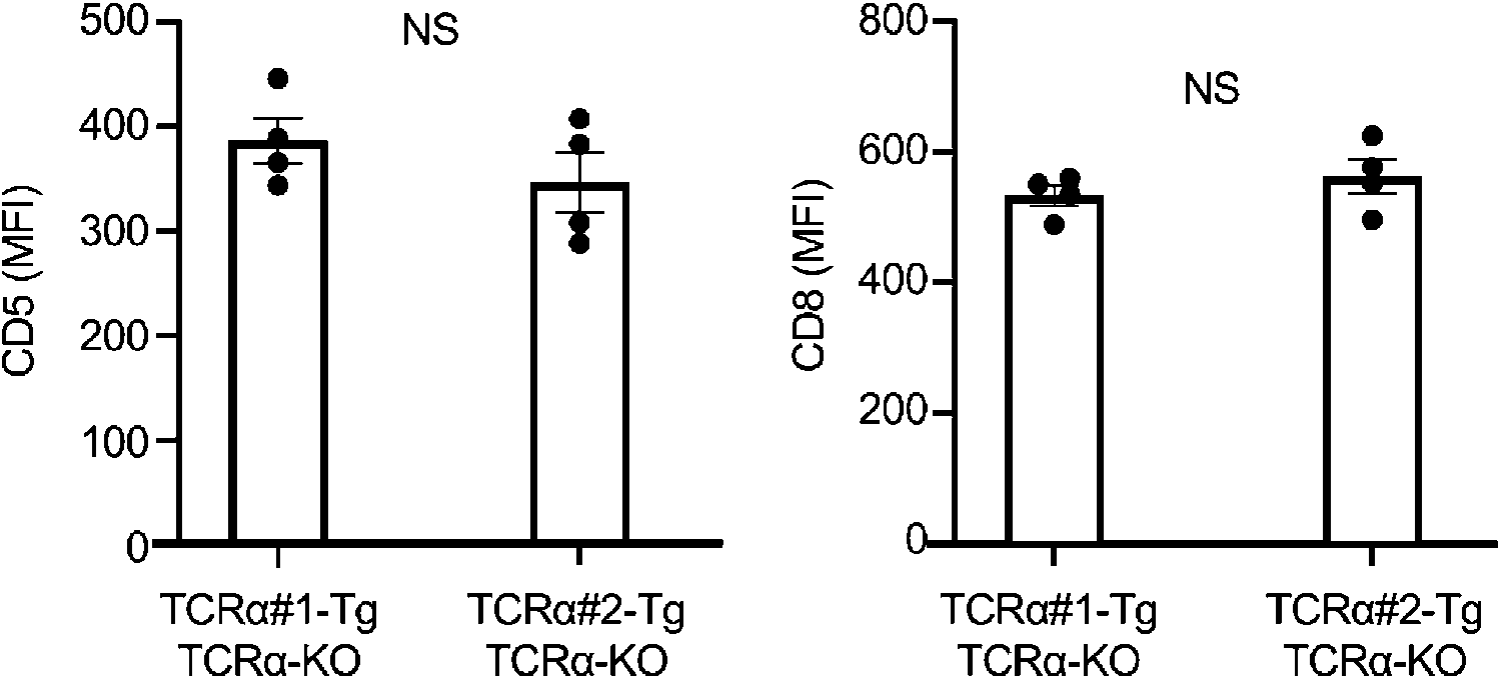
**CD5 and CD8 expression in CD4**^**+**^**CD8**^**+**^** CD69**^**+**^**CCR7**^**−**^** thymocytes.** Mean fluorescence intensity (MFI; means and SEMs, *n* = 4 in two independent measurements) of cell-surface CD5 and CD8 expression in CD4^+^CD8^+^ CD69^+^CCR7^−^ Vα8.3^+^ thymocytes from TCRα#1-transgenic, TCRα-deficient mice and TCRα#2-transgenic, TCRα-deficient mice (by unpaired *t* test).

It is also interesting to note that the cell-surface expression of CD5 and CD8 at the CD4^+^CD8^+^ CD69^+^CCR7^−^ stage was equivalent—and not significantly different—between β5t-dependent TCRα#1-expressing thymocytes and β5t-independent TCRα#2-expressing thymocytes (Fig. S4), suggesting that the thymoproteasome dependency of these TCRα#1- and TCRα#2-expressing thymocytes is not readily correlated with the TCR affinity to peptide–MHC-I ligands that induce positive selection in the thymic cortex (Nitta et al., 2010; Xing et al., 2013; Takada et al., 2015).

Furthermore, it should be additionally pointed out that TCRα#2-expressing thymocytes give rise to mature CD8^+^ T cells preferentially in the absence of β5t, instead of being deleted in the thymus. These data argue against the possibility that the function of β5t is to avoid the negative selection of thymocytes by creating a difference in MHC-I–associated peptides between cTECs and other antigen-presenting cells.

### The thymoproteasome optimizes CD8^+^ T cell production independent of mTECs and thymic medulla

**Figure 4.**
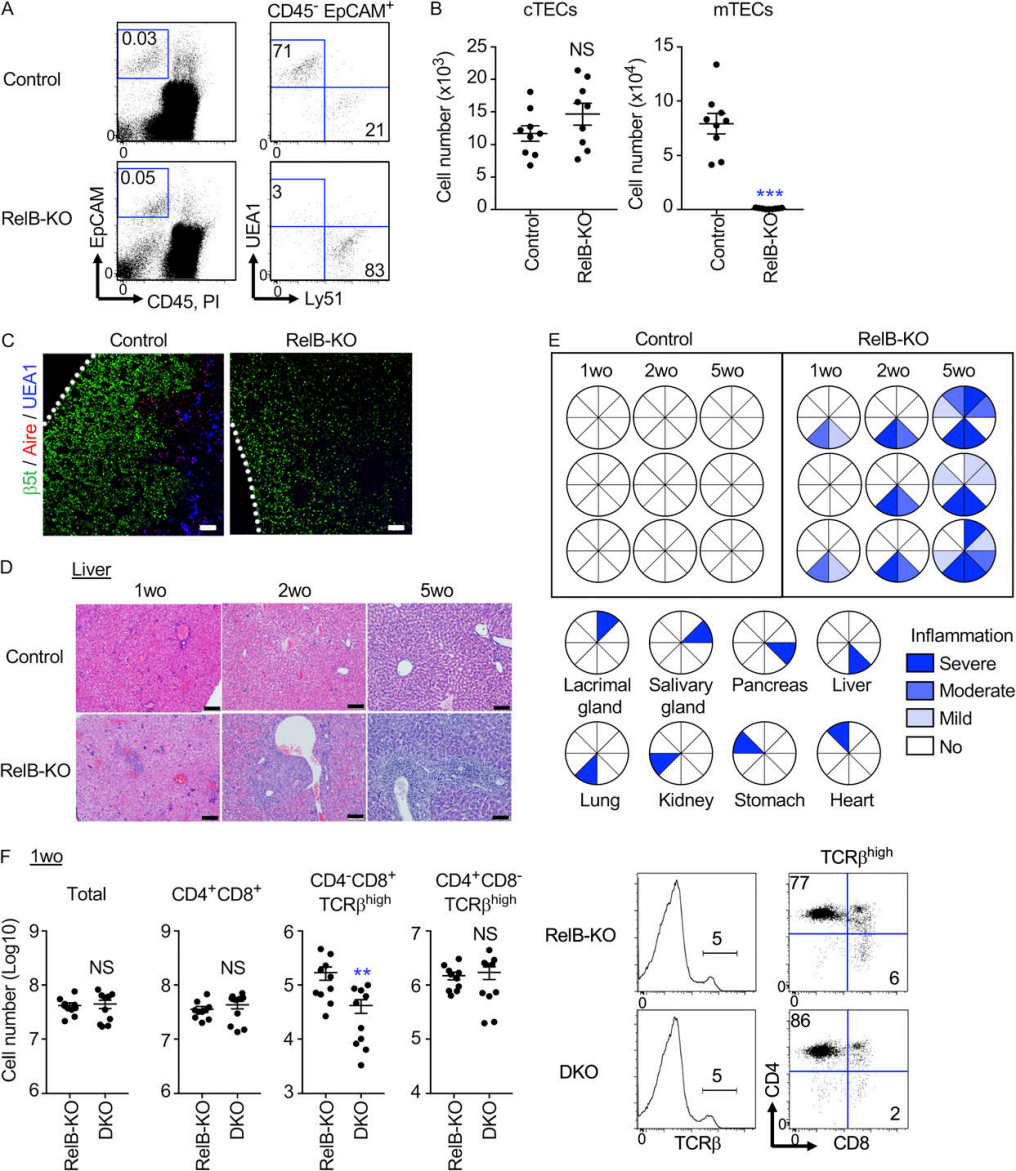
**The thymoproteasome optimizes CD8^+^ T cell production in the absence of the thymic medulla.** **(A)** Flow cytometric analysis of Liberase-digested thymic cells from 2-wk-old relB-deficient mice. Dot plots show EpCAM and CD45 expression in total thymic cells (left) and UEA1 reactivity and Ly51 expression in CD45^−^EpCAM^+^-gated epithelial cells (right). Numbers in dot plots indicate the frequency of cells within the indicated area. **(B)** Cell number (means and SEMs, *n* = 9 in four independent measurements) of CD45^−^EpCAM^+^UEA1^−^Ly51^+^ cTECs and CD45^−^EpCAM^+^ UEA1^+^Ly51^−^ mTECs. ***, P < 0.001 (by unpaired *t* test with Welch’s correction). **(C)** Immunofluorescence analysis of thymic sections from 2-wk-old relB-KO mice. β5t (green), UEA1 reactivity (blue), and Aire (red). Representative data from three independent experiments are shown. Scale bars, 100 µm. **(D)** Hematoxylin and eosin–stained liver sections from indicated mice. Representative results of at least three independent experiments. Scale bar, 100 µm. **(E)** Inflammation grades in indicated tissues from control mice and relB-KO mice. **(F)** Flow cytometric analysis of thymocytes from relB-KO mice and relB/β5t-double KO (DKO) mice at 1 wk old. Cell number (means and SEMs, *n* = 10 in seven independent measurements) of indicated thymocyte populations. **, P < 0.01 (by unpaired *t* test with Welch’s correction). Histograms for TCRβ expression in propidium iodide (PI)^−^ viable cells and dot plots for CD8 and CD4 expression in PI^−^ TCRβ^high^ cells are also shown. Numbers indicate the frequency of cells within the indicated area. wo, wk old.

**Figure S5.**
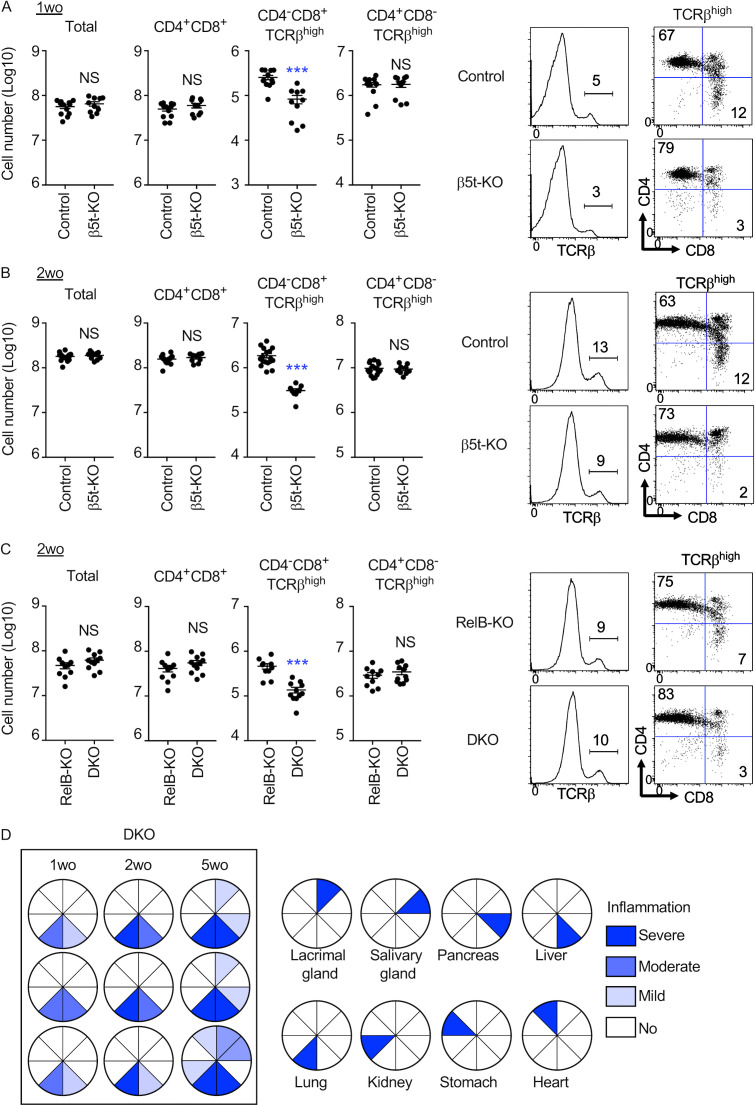
**Thymocyte profiles and tissue inflammation grades in β5t-deficient and relB-deficient mice. (A–C)** Flow cytometric analysis of thymocytes from indicated mice at 1 wk old (A) and 2 wk old (B and C). Cell number (means and SEMs, *n* = 9–17 in five to seven independent measurements) of indicated thymocyte populations. ***, P < 0.001 (by unpaired *t* test with Welch’s correction). Histograms for TCRβ expression in PI^−^ viable cells and dot plots for CD8 and CD4 expression in PI^−^ TCRβ^high^ cells are also shown. Numbers indicate the frequency of cells within the indicated area. **(D)** Inflammation grades in indicated tissues from relB/β5t-double KO (DKO) mice. wo, wk old.

To further analyze the contribution of the thymic microenvironments to the thymoproteasome-dependent CD8^+^ T cell optimization, we next examined CD8^+^ T cell development in mice that were deficient in relB, a transcription factor essential for the development of mTECs and the thymic medulla (Burkly et al., 1995; Weih et al., 1995). The number of mTECs was markedly diminished, whereas the cTEC number was unimpaired in the thymus of relB-deficient mice (Fig. 4, A and B). Indeed, the thymus of relB-deficient mice lacked the medullary region that contained Aire^+^ mTECs, whereas the cortical region containing β5t^+^ cTECs was unimpaired (Fig. 4 C). The lack of mTECs and the thymic medulla reslted in the defective establishment of self-tolerance in T cells and therefore the onset of autoimmune disease within 5 wk postnatally (Fig. 4, D and E; Burkly et al., 1995; Weih et al., 1995). Autoimmune inflammation in relB-deficient mice was mildly detectable as early as 1 wk old and became moderate at 2 wk old before progressing to severe damage in many tissues by 5 wk old (Fig. 4, D and E). In accordance with and in response to the multiorgan inflammation (Weih et al., 1995; O’Sullivan et al., 2018), thymic atrophy as measured by the reduced number of total thymocytes was detected in relB-deficient mice mildly at 1 wk old and moderately at 2 wk old (Fig. 4 F and Fig. S5, A–C).

Importantly, we found that the development of CD4^−^CD8^+^ TCRβ^high^ thymocytes was significantly impaired in β5t-deficient thymus even in the absence of relB. The impairment was detected at 1 wk old and 2 wk old (Fig. 4 F and Fig. S5, A–C), indicating that the thymoproteasome optimizes CD8^+^ T cell production even in the absence of mTECs and the thymic medulla, which are dependent on relB. The reduction in the number of CD4^−^CD8^+^ TCRβ^high^ thymocytes as a result of β5t deficiency was comparable (28.1 ± 5.6%, *n* = 7; and 28.8 ± 6.0%, *n* = 6) and not significantly different (P > 0.05) between the absence and presence of relB, suggesting that there is no significant contribution of relB-dependent medullary negative selection in the β5t-dependent production of CD8^+^ T cells.

It is additionally interesting to point out that the progressive multiorgan inflammation in relB-deficient mice at 1, 2, and 5 wk of age was detected equivalently in β5t-deficient relB-deficient mice (Fig. S5 D), indicating that the onset of autoimmune disease caused by the loss of relB is independent of the thymoproteasome and thymoproteasome-dependent CD8^+^ T cells.

### The thymoproteasome optimizes CD8^+^ T cell production independent of additional antigen-presenting cells

**Figure 5.**
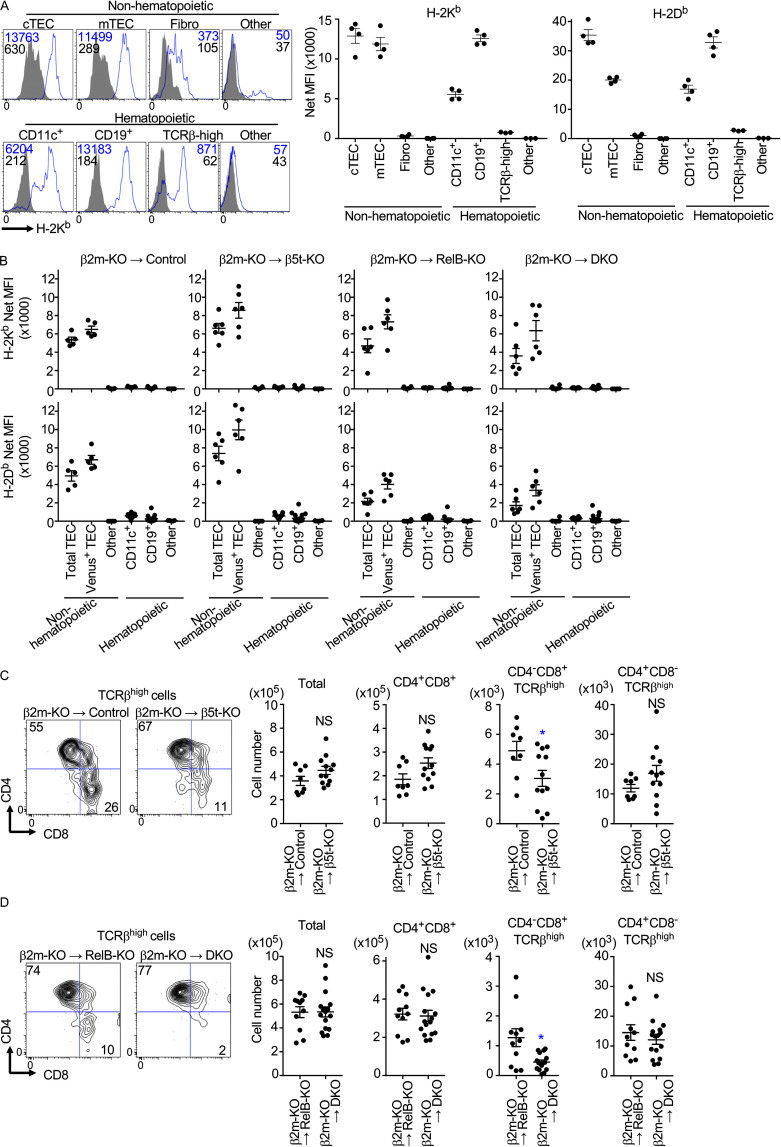
**The thymoproteasome optimizes CD8^+^ T cell production in the absence of additional antigen-presenting cells.** **(A)** MHC-I expression in indicated cell populations from 2-wk-old B6 mice. Histograms show the expression of H-2K^b^ (blue) and background fluorescence (shaded) in cTECs, mTECs, PDGFRβ^+^CD45^−^EpCAM^−^ fibroblasts, PDGFRβ^−^CD45^−^EpCAM^−^ other nonhematopoietic cells, CD11c^+^ DCs, CD19^+^ B cells, TCRβ^high^ thymocytes, and CD11c^−^ CD19^−^TCRβ^negative-low^ other hematopoietic cells. Numbers in histograms indicate median fluorescence intensity (MFI) for H-2K^b^ (blue) and background (black). Plots (means and SEMs, *n* = 3–4 in three independent measurements) show net MFI values for H-2K^b^ and H-2D^b^. **(B–D)** dGuo-treated fetal thymuses from control mice, β5t-KO mice, relB-KO mice, and double KO (DKO) mice were reconstituted with β2m-KO fetal thymocytes cultured for 7 d. **(B)** Net MFI values (means and SEMs, *n* = 3–16 in at least two independent measurements) for H-2K^b^ (top) and H-2D^b^ (bottom) in indicated cell populations are shown. Heterozygous or homozygous β5t-Venus knock-in knock-out allele was included, so that Venus^+^ TECs represented β5t-expressing cTECs. **(C and D)** Contour plots for CD8β and CD4 expression in PI^−^ TCRβ^high^ thymocytes from indicated fetal thymus organ cultures are shown. Numbers in plots indicate the frequency of cells within the indicated area. Cell numbers (means and SEMs; *n* = 8–12 in C, *n* = 11–16 in D in four independent measurements) of indicated thymocyte populations measured in four independent experiments are plotted. *, P < 0.05 (by unpaired *t* test with Welch’s correction).

Antigen-presenting cells in the thymus include various hematopoietic and nonhematopoietic cells. In addition to cTECs and mTECs, CD11c^+^ DCs and CD19^+^ B cells abundantly express MHC-I molecules and play an important role in antigen presentation for the establishment of T cell self-tolerance in the thymus (Fig. 5 A; Gallegos and Bevan, 2004; Fujihara et al., 2014; Yamano et al., 2015). On the contrary, other thymic cells, including thymocytes and fibroblasts, expressed limited amounts of MHC-I molecules (Fig. 5 A), playing no or a minimal role in inducing the MHC-I–dependent positive selection of CD8^+^ T cells (Anderson et al., 1994; Laufer et al., 1996; Chidgey et al., 1998; Lilic, et al., 2002), although thymocytes could induce the MHC-I–dependent negative selection of neighboring thymocytes (Schönrich et al., 1993; Melichar et al., 2015). To examine the involvement of hematopoietic cells, including DCs and B cells, in thymoproteasome-dependent CD8^+^ T cell production, embryonic thymus lobes were treated with 2′-deoxyguanosine (dGuo) to deplete hematopoietic cells, including thymocytes, DCs, and B cells, and were reconstituted with immature hematopoietic cells isolated from β2-microglobulin (β2m)–deficient mice, which lacked the surface expression of peptide–MHC-I complexes (Koller et al., 1990). Indeed, DCs, B cells, and other hematopoietic cells in the reconstituted thymus were defective in the surface expression of MHC-I (Fig. 5 B), and the surface MHC-I expression in the reconstituted thymus was limited to TECs, including β5t^Venus+^ cTECs (Fig. 5 B). We found that the thymus reconstituted with β2m-deficient hematopoietic cells still was significantly impaired in the development of CD4^−^CD8^+^ TCRβ^high^ thymocytes in β5t-deficient thymus, even in the absence of relB-dependent mTECs (Fig. 5, C and D). The reduction in cell number by the loss of β5t was specific to CD4^−^CD8^+^ TCRβ^high^ thymocytes, as the numbers of CD4^+^CD8^+^ and CD4^+^CD8^−^ TCRβ^high^ thymocytes were comparable in the absence or presence of β5t (Fig. 5, C and D). We also noticed that the number of CD4^−^CD8^+^ TCRβ^high^ thymocytes was reduced by the loss of relB in the thymus (Fig. 5, C and D), which agreed with the reduction of H-2D^b^ expression by β5t^+^ cTECs in relB-deficient thymus (Fig. 5 B).

These results indicate that the thymoproteasome expressed by cTECs optimizes CD8^+^ T cell production even in the absence of additional antigen-presenting cells, including mTECs, DCs, and B cells, and in the absence of the thymic medullary microenvironment. The results also reveal that the function of relB in TECs is not limited to the development of mTECs but includes the elevation of cell-surface MHC-I expression in cTECs.

### Thymoproteasome-dependent positive selection of cortical thymocytes operates independent of negative selection

**Figure 6.**
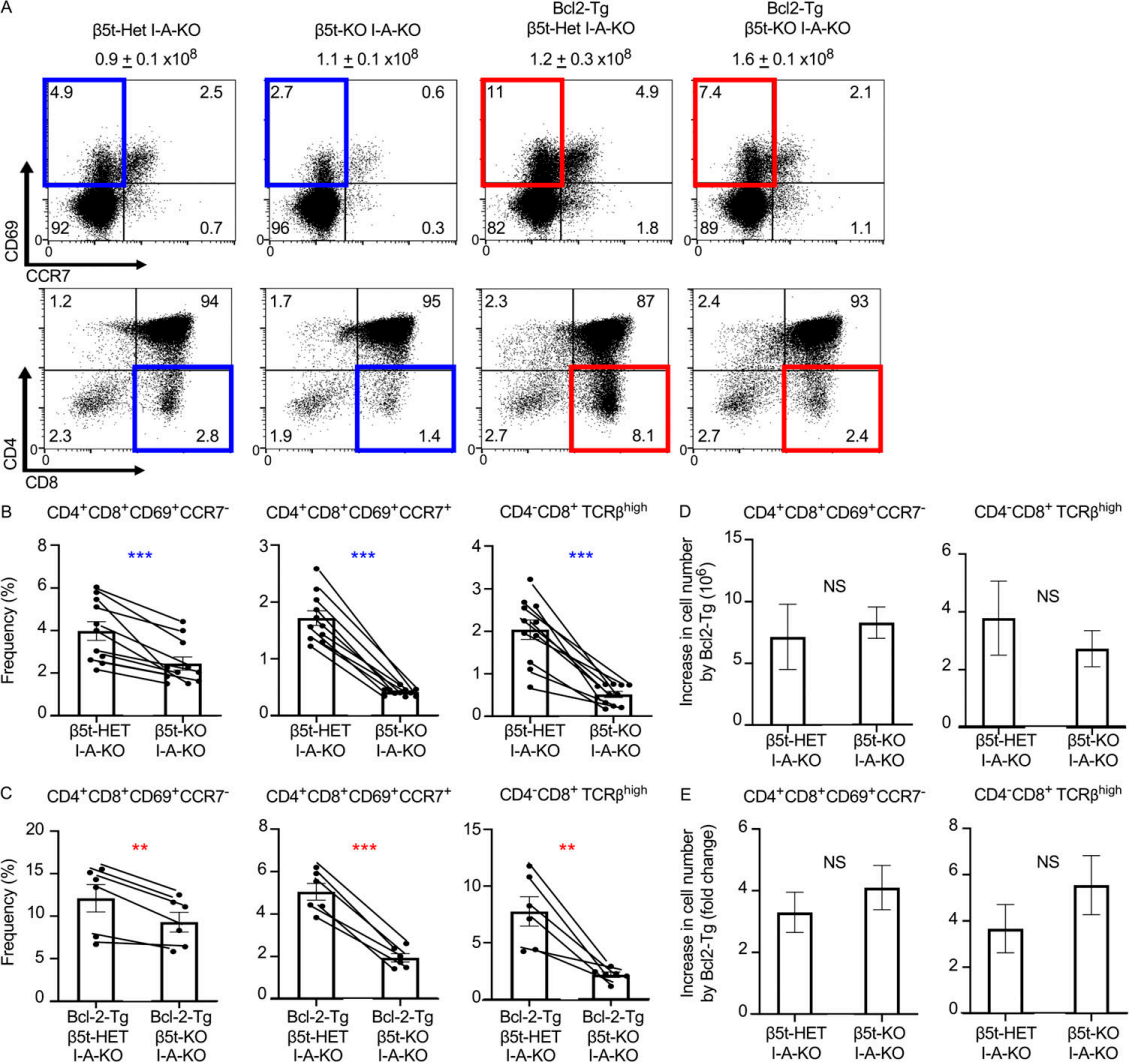
**Contribution of negative selection in thymoproteasome-dependent production of CD8^+^ T cells. (A)** Flow cytometric analysis of viable thymocytes from indicated mice at 4–10 wk old. Total viable cell numbers (means and SEMs, *n* = 8–9 in seven independent measurements) are listed. The frequency of cells within the indicated area is also shown. **(B and C)** Generation of indicated thymocyte subpopulations in Bcl2-Tg–negative mice (B) and Bcl2-Tg–positive mice (C) is shown as the frequency of cell numbers relative to the numbers of CD4^+^CD8^+^ CD69^−^CCR7^−^ immature thymocytes. **(D and E)** Differences (D) and fold changes (E) in cell numbers of indicated thymocyte subpopulations between Bcl2-Tg–positive and –negative mice are shown. **, P < 0.01; ***, P < 0.001 (by paired *t* test).

Our results so far indicate that the thymoproteasome expressed by cTECs affects the TCR repertoire of cortical thymocytes and optimizes CD8^+^ T cell production independent of additional antigen-presenting cells. These results argue against the contribution of the thymic medulla–dependent mechanism in the thymoproteasome-mediated optimization of CD8^+^ T cells. However, the negative selection of thymocytes is detected even in the thymic cortex (Stritesky et al., 2013), and it is controversial how the negative selection contributes to thymoproteasome-dependent T cell development (Xing et al., 2013; Kincaid et al., 2016). We finally examined the contribution of the negative selection to thymoproteasome-dependent CD8^+^ T cell production in the thymic cortex. To assess this, we crossed β5t-deficient mice to proximal Lck promoter–driven Bcl-2 transgenic mice, in which thymocyte negative selection is prevented by inhibiting proapoptotic BH3-only family proteins, including Bim and Puma (Sentman et al., 1991; Pobezinsky et al., 2012). The mice were additionally crossed to I-Aβ–deficient mice, which lack cell-surface MHC-II expression and thereby lack TCR-mediated selection and development of CD4^+^ lineage T cells (Cosgrove et al., 1991) to specifically detect MHC-I–dependent selection and development of CD4^+^CD8^+^ thymocytes toward CD8^+^ lineage T cells (Fig. 6 A). In the absence of MHC-II, and therefore the absence of CD4^+^ lineage thymocyte selection events, we found that the absence of β5t significantly decreased the generation of positively selected thymocytes, even at the earliest CD4^+^CD8^+^ CD69^+^CCR7^−^ cortical stage (Fig. 6, A and B), indicating that the thymoproteasome optimizes the MHC-I–dependent positive selection of cortical thymocytes. We further found that the lack of β5t decreased positively selected CD8^+^ lineage thymocytes at the CD4^+^CD8^+^ CD69^+^CCR7^−^ cortical stage equivalently in the presence or absence of anti-apoptotic Bcl-2 (Fig. 6, B and C). These results indicate that the thymoproteasome optimizes the MHC-I–dependent positive selection of cortical thymocytes before migration to the thymic medulla and independent of apoptosis-mediated negative selection.

The increased cell numbers that resulted from Bcl-2–mediated prevention from any potential negative selection in CD4^+^CD8^+^ CD69^+^CCR7^−^ newly selected cortical thymocytes were 7.1 ± 2.6 × 10^6^ (3.7-fold increase) and 8.3 ± 1.3 × 10^6^ (4.1-fold increase) in the presence and absence of β5t, respectively (Fig. 6, D and E), showing that the β5t-deficient thymus exhibits no significant increase in the number of cells undergoing the deletion in the thymic cortex. Accordingly, there was a 2.7 ± 0.6 × 10^6^ (5.5-fold) greater abundance of CD4^−^CD8^+^TCRβ^high^ mature thymocytes in Bcl-2 transgenic β5t-deficient mice than in β5t-deficient mice compared with a greater abundance of 3.8 ± 1.3 × 10^6^ (3.7-fold) in Bcl-2 transgenic mice relative to that in WT mice (Fig. 6, D and E), showing that negative selection equivalently, and not differently, impacts the generation of CD4^−^CD8^+^ mature thymocytes in the presence or absence of β5t. These results indicate that the thymoproteasome-dependent positive selection of cortical thymocytes operates independent of apoptosis-mediated negative selection.

## Discussion

Our results indicate that β5t-containing thymoproteasome expressed by cTECs shapes the TCR repertoire of positively selected thymocytes in the thymic cortex before the migration to the thymic medulla. Our results also indicate that the thymoproteasome optimizes CD8^+^ T cell production independent of the thymic medulla; independent of additional antigen-presenting cells, including mTECs, DCs, and B cells; and independent of the apoptosis-mediated negative selection of developing thymocytes. Thus, the thymic medulla–dependent and/or apoptosis-mediated negative selection of positively selected thymocytes (Kincaid et al., 2016; Tomaru et al., 2019), a possibility originally described in 1980s (Marrack and Kappler, 1987; Kourilsky and Claverie, 1989), cannot readily serve as the basis for the thymoproteasome-mediated optimization of CD8^+^ T cell production.

Instead, our results reveal that CD4^+^CD8^+^ thymocytes that express the full-length TCRα chain derived from CD8^+^ T cells are destined to develop preferentially into CD8^+^ T cells rather than CD4^+^ T cells. These findings suggest that the full-length TCRα sequence, including the CDR3 sequence, influences the MHC restriction specificity of TCR recognition by T cells. Importantly, we found that the full-length TCRα#1-expressing CD8^+^ T cells preferentially develop in the thymoproteasome-expressing WT thymus, whereas the TCRα#2-expressing CD8^+^ T cells develop independent of the thymoproteasome and preferentially in the β5t-deficient thymus. These results, surprisingly, indicate that the full-length TCRα variable region structure, including the CDR3 sequence, is hardwired with the thymoproteasome-mediated optimization of CD8^+^ T cell production. Thus, our results reveal that the thymoproteasome connects cortical positive selection with the TCR repertoire of CD8^+^ T cells, independent of negative selection.

A recent report by Kincaid et al. (2016) demonstrated that the majority of CD8^+^ T cells positively selected in mice deficient in β1i, β2i, β5i, and β5t (4KO mice) failed to pass the negative selection checkpoint in the thymus. Their results showed that the magnitude of the rescue of CD4^−^CD8^+^ mature thymocytes by the loss of the proapoptotic protein Bim was much greater (22.7-fold over 5.0-fold; statistically significant difference) in the 4KO thymus than in the WT thymus. Based on these results, the report supports the hypothesis that most developing CD8^+^ T cells need to be selected on different peptides at positive selection and negative selection (Kincaid et al., 2016). In contrast, our results show that the magnitude of the rescue of CD4^−^CD8^+^ mature thymocytes by transgenic Bcl-2 expression, which inhibits proapoptotic BH3-only family proteins, not limited to Bim but included Puma (Pobezinsky et al., 2012), was essentially equivalent (5.5-fold over 3.7-fold; no statistically significant difference) in the thymus of β5t-deficient mice and control mice. These results indicate that the negative selection–dependent mechanism has no appreciable role in the thymoproteasome-mediated optimization of CD8^+^ T cell production. These results further suggest that the failure to pass the negative selection detected in 4KO mice is not primarily due to the loss of β5t-containing thymoproteasome in cTECs, but rather results from the loss of the immunoproteasome and its components β1i, β2i, and β5i, which are highly expressed in mTECs and DCs in the thymus (Nil et al., 2004).

The contribution of the thymoproteasome in the negative selection–dependent mechanism has also been supported in the recent analysis of β5t-transgenic, β5i-deficient mice that were engineered to ubiquitously express β5t but not β5i (Tomaru et al., 2019). They reported that the generation of CD8^+^ T cells was impaired in β5t-transgenic, β5i-deficient mice; however, those systemic β5t-transgenic mice were metabolically aberrant and showed weight loss due to the systemic reduction of chymotrypsin-like proteasomal activity (Tomaru et al., 2012). The study also showed that MHC-I expression was reduced in those β5t-transgenic, β5i-deficient animals (Tomaru et al., 2019). Thus, the aberrant thymocyte development in their β5t-transgenic, β5i-deficient mice may not be simply due to the loss of the MHC-I–associated peptide switching, but likely results from the combination of multiple abnormalities, including systemic aberrancy in the metabolism, in those transgenic mice.

Our results further demonstrate that the increases in absolute cell numbers that Bcl-2 prevented from negative selection in CD4^+^CD8^+^ CD69^+^CCR7^−^ newly selected cortical thymocytes were equivalent in the presence and absence of β5t. These results agree with previous results demonstrating that the percentage of CD4^+^CD8^+^ CD69^+^TCRβ^+^ thymocytes derived from Bim-deficient bone marrow cells showed a similar rescue of cells from negative selection in the presence and absence of β5t (Xing et al., 2013). These results further support that the thymoproteasome-mediated optimization of CD8^+^ T cell production is not merely due to the thymoproteasome-dependent generation in cTECs of MHC-I–associated self-peptides that are distinct from those peptides in mTECs, DCs, and other antigen-presenting cells.

It should be noted, however, that our results do not entirely rule out the possibility that the negative selection by additional antigen-presenting cells contributes to the β5t-dependent production of CD8^+^ T cells. Indeed, our data show that the number of CD4^−^CD8^+^ TCRβ^high^ thymocytes in the β5t-deficient, relB-deficient thymus was 39.5 ± 9.4% (*n* = 4) of that in the relB-deficient thymus in fetal thymus organ culture where hematopoietic cells lacked MHC-I molecules (Fig. 5 D), whereas the number of CD4^−^CD8^+^ TCRβ^high^ thymocytes in the β5t-deficient, relB-deficient thymus was 28.8 ± 6.0% (*n* = 6) of that in the relB-KO thymus (Fig. 4 F) in the presence of MHC-I molecules on hematopoietic cells. Although these cell number ratios do not show a statistically significant difference (P > 0.05), it is possible that MHC-I molecules expressed by hematopoietic cells play a minor role in the β5t-dependent optimization of CD8^+^ T cell production.

Finally, our deep-sequencing analysis of TCRα and TCRβ mRNAs expressed in CD8^+^ T cells isolated from four β5t-deficient mice and four control mice indicated that the repertoire of TCRα and TCRβ sequences in CD8^+^ T cells from β5t-deficient mice were similarly diverse compared with that from control mice, as estimated by the Shannon-Weaver diversity index (Fig. 1 B). Interestingly, however, the number of full-length TCR sequences shared in the four individual mice was higher in β5t-deficient mice than control mice (Fig. 1 E). The basis for these differences is unclear, although it is possible that β5t-dependent positive selection in normal thymic cortex may somehow restrict TCR specificities to form a functionally competent TCR repertoire shared by individual mice.

In conclusion, our results support a direct role of the thymoproteasome intrinsic to the positive selection in the thymic cortex, governing the generation of the TCR repertoire of CD8^+^ T cells. The thymoproteasome may contribute to the preferential production of MHC-I–associated self-peptides that carry structural advantages to interact with TCR structures, including the structure in the TCRα#1 V-J sequence, that are positively selected into CD8^+^ T cells. The present results highlight the importance of the future identification of self-peptide–MHC-I complexes displayed by WT and thymoproteasome-deficient mouse cTECs. Recent analysis of thymic epithelial cells in genetically engineered mice that carry an enlarged thymus (Ohigashi et al., 2019) has suggested the feasibility of biochemically identifying β5t-dependent MHC-I–associated peptide sequences freshly isolated from mouse cTECs, which we believe would benefit our understanding of the fundamental basis for the thymus-dependent positive selection of T cells.

## Materials and methods

### Mice

C57BL/6 (B6) were obtained from The Jackson Laboratory and SLC Japan. B6 background mice deficient in β5t (Murata et al., 2007), relB (Burkly et al., 1995), I-A^b^β (Cosgrove et al., 1991), β2m (Koller et al., 1990), and TCRα (Mombaerts et al., 1992), as well as transgenic for Bcl-2 (Sentman et al., 1991), were bred in our animal facility. All mouse strains were backcrossed at least 10 times to B6 mice. Littermates were used as control animals. All mouse experiments were performed with consent from the Animal Experimentation Committee of the University of Tokushima (T2019–62) and from the Animal Care and Use Committee of the National Cancer Institute (ASP 18–431 and EIB-076–2).

### TCRα-transgenic mice

Full-length TCRα sequences were cloned into human CD2-transgenic vector (Zhumabekov et al., 1995). TCRα-transgenic mice were bred to TCRα-deficient mice. Three or four lines of individual TCRα-transgenic mice produced essentially identical results in thymocyte development.

### Bone marrow chimeras

Bone marrow cells were depleted of T cells using anti-Thy1.2 MACS Microbeads (Miltenyi). Mice were lethally irradiated (9.5 Gy) and reconstituted with T cell–depleted bone marrow cells (2.5 × 10^6^ cells). Mice were analyzed 5–8 wk after reconstitution.

### Fetal thymus organ culture

Fetal thymus lobes isolated on embryonic day (E) 15.5 were organ cultured for 5 d on sponge-supported Nucleopore filters (Whatman) placed on RPMI 1640–based complete cell culture medium containing 1.35 mM dGuo. dGuo-treated thymus lobes were washed with PBS for 30 min twice. Fetal thymocytes isolated from E15.5 β2m-deficient mice were cultured with dGuo-treated thymus lobes (1 lobe/well) in a hanging drop (20 μl/well) in Terasaki plate for 24 h. Lobes were transferred onto freshly prepared Nucleopore membranes and organ cultured for 7 d (Ramsdell et al., 2006).

### Flow cytometric analysis

For the analysis of nonhematopoietic thymic cells, minced thymuses were digested with 0.5 U per ml Liberase (Roche) and 0.02% DNase I (Roche). Single-cell suspension was stained for the expression of platelet-derived growth factor receptor b (clone APB5; eBioscience), CD45 (clone 30-F11; eBioscience), EpCAM (clone G8.8; BioLegend), Ly51 (clone 6C3; BioLegend), H-2K^b^ (clone AF6-88.5; BioLegend), and H-2D^b^ (clone 28–14-8; eBioscience), and for the reactivity with UEA1 (Vector Laboratories). For the analysis of thymocytes and spleen cells, cells were stained for the expression of CD4 (clone RM4-5; eBioscience), CD8α (clone 53–6.7; eBioscience), CD8β (clone 53–5.8; BD PharMingen), CD5 (clone 53–7.3; BD Biosciences), CD11c (clone N418; BioLegend), CD19 (clone 6D5; BioLegend), TCRβ (clone H57-597; BioLegend), CD69 (clone H1.2F3; BioLegend), Vα8.3 (clone B21.14; BioLegend), CD45.2 (clone 104; BD PharMingen), CD45.1 (clone A20; BD Biosciences), CD44 (clone IM7; BioLegend), CD122 (clone TM-β1; BioLegend), CD62L (clone MEL-14; BD Horizon), and CCR7 (clone 4B12; Invitrogen). Biotinylated antibodies were detected by Pacific Blue–conjugated or Alexa 594–labeled streptavidin (Invitrogen). Flow cytometric analysis was performed on BD LSR Fortessa and FACSVerse (BD Biosciences). For the analysis of the intracellular expression of TCRβ, thymocytes were stained for cell-surface proteins and treated with Fixation/Permeabilization Buffer (Invitrogen), and intracellular staining was performed in Permeabilization Buffer (Invitrogen) and normal mouse serum (Jackson ImmunoResearch).

### Immunofluorescence analysis

Thymus tissues were fixed with 4% (g/vol) paraformaldehyde and embedded in optimum cutting temperature compound (Sakura Finetek). Frozen thymuses were sliced into 10-µm-thick sections and stained with antibodies specific for β5t (Murata et al., 2007) and Aire (clone 5H12; Invitrogen). Sections were also stained for reactivity with UEA1 (Vector Laboratories). Images were visualized and analyzed with a TCS SP8 (Leica) confocal laser scanning microscope.

### Pathology

Histologic grading of inflammatory lesions in hematoxylin and eosin–stained sections of all tissues fixed with 10% phosphate-buffered formalin (pH 7.2) was performed as described previously (Kohashi et al., 2008). Mild inflammation indicates that one to five foci composed of >20 mononuclear cells per focus were seen, moderate inflammation indicates that more than five such foci were seen, but without significant parenchymal damage, and severe inflammation indicates degeneration of parenchymal tissue.

### Deep sequencing analysis of TCR repertoire

10^6^ CD8^+^ T cells were sorted from lymph nodes and spleens in β5t^+/−^ and β5t^−/−^ mice (6–7-wk-old, male, *n* = 4 individual mice/group, >98% purity). cDNAs were amplified for TCRα or TCRβ genes with the ligation of a universal adaptor to the leader sequence of variable regions, which permitted effective and reproducible amplification of all TCR genes without bias due to variable sequences (Repertoire Genesis; Tsuruta et al., 1993). Large-scale, pair-ended sequencing was performed by using Illumina MiSeq. More than 2 × 10^6^ on average of total read sequences at 400–500 bp were analyzed.

### Quantitative RT-PCR analysis

Total cellular RNA was reverse transcribed with the PrimeScript Reverse transcription (Takara). PCR was performed by using ExTaq (Takara), and the PCR products were used for nested quantitative real-time PCR by using SYBR Premix ExTaq (Takara) and a StepOnePlus Real-Time PCR System (Applied Biosystems). The amplified products were confirmed to be single bands by gel electrophoresis.

### Single-cell RNA sequencing analysis

Thymocytes were stained for the expression of CD4, CD8α, TCRβ, CD69, and CCR7, and were sorted on FACS Aria II (BD Biosciences). Sorted cells were labeled with TotalSeq C hashtag oligos specific for CD45 (30-F11; BioLegend) and MHC-I (M1/42; BioLegend). Single-cell 5′ transcriptomic profiling was performed by using 10× Genomics system, and the data were analyzed by using RStudio software.

### Data availability

Deep sequencing data of TCR genes have been deposited in The DNA Data Bank of Japan (https://www.ddbj.nig.ac.jp) with the accession no. DRA011284. Single-cell RNA sequencing data have been deposited in NCBI Gene Expression Omnibus (https://www.ncbi.nlm.nih.gov/geo) with the accession no. GSE164895.

### Online supplemental material

Fig. S1 shows the use of TCRα and TCRβ V regions detected in CD8^+^ T cells from β5t-deficient mice. Fig. S2 shows flow cytometric profiles of spleen cells from bone marrow chimera mice. Fig. S3 shows flow cytometric profiles of CD62L, CD69, CCR7, Vα8.3, and TCRβ expressed by thymocyte subpopulations from bone marrow chimera mice. Fig. S4 shows mean fluorescence intensity of CD5 and CD8 expression in CD4^+^CD8^+^ CD69^+^CCR7^−^ Vα8.3^+^ thymocytes from TCRα#1-transgenic and TCRα#2-transgenic TCRα-deficient mice. Fig. S5 shows cell numbers and flow cytometric profiles of thymocytes as well as inflammation grades of the tissues from β5t-deficient and relB-deficient mice. Table S1 lists the top five unique TCRα and TCRβ sequences detected in CD8^+^ T cells from β5t-deficient mice.

## Supplementary Material

Table S1 shows the top five unique TCRα and TCRβ sequences detected in CD8^+^ T cells from β5t^+/−^ (Het) mice and β5t^−/−^ (KO) mice.
